# Edgar Park, D.D.S.

**Published:** 1893-03

**Authors:** Wm. Conrad


### Edgar Park, D.D.S.

At a meeting of the St. Louis Dental Society, held January 17th, 1893, the committee appointed to prepare a memorial and resolutions on the death of Dr. Edgar Park, formerly an active member of this Society, submitted the following report, which was adopted:

### IN MEMORIAM.

Died at Middleton, New York, August 12th, 1892, Dr. Edgar Park, in the fifty-second year of his age.

Dr. Park was born in Wainfleet, County of Welland, Ontario, April 21, 1840. His family were English people. Up to the time of his leaving home his education was only such as was obtained from the common or public schools. He left his home at the age of sixteen with but a scanty wardrobe and twenty dollars, bound for Texas, but, owing to his financial condition, stopped at Chicago, where he obtained employment, and by taking a course in Bryant & Stratton's Business College made some advance in education and position. Through the influence and association with his uncle, Dr. Park of Chicago, with whom he lived, he became deeply interested in the study of medicine, and attended a regular course at Rush Medical College. Later his attention was directed to the special branch of dentistry, and he adopted it as his profession, entering upon a course of study at the Ohio Dental College in the year 1864, and graduating at the Missouri Dental College in 1869.

He was associated in practice with Dr. W. W. Alport, of Chicago, for a short time, after which he came to St. Louis and took a position as assistant to Dr. C. W. Spalding in 1865. In 1867 he became associated with Dr. H. J. McKellops, and remained with him until 1870 when he opened an office of his own. He soon secured a lucrative practice, and was highly esteemed socially and professionally. In 1873 he was happily married to Mary C. Fisk, an accomplished and lovable woman, daughter of General Clinton B. Fisk.

He continued the practice of his profession until March, 1884, when failing health compelled him to retire, and, after an illness of eight years, died at Middleton, New York, August 12, 1892.

His wife and five children (four daughters and one son) are left to mourn the death of a devoted and affectionate husband and father. Dr. Park was devoted to his profession, and was often heard to remark that "If I were worth a million, I would never give up the practice."

He was a progressive man, and ambitious to see the professional standard raised and honored; was foremost with those who labored to advance the profession by filling its ranks with honorable, capable men. He was a self-made man in every respect—a man of great natural resources and exquisite literary taste, and extravagantly fond of art, and always longed for the moment when he could have time and means to gratify his taste in this direction.

He was a devoted professional brother, interesting, sociable, kind and charitable, an active worker in the St. Louis Dental Society, always ready to do his share in whatever position he was called to act. He received the highest honors in the gift of the St. Louis Dental Society. He was a member of the American Dental Association, and for one year Recording Secretary. Also a member of the Missouri State Dental Association.

He was, at one time, a member of the Illinois Dental Society.

Dr. Park was a careful, painstaking, conscientious operator of acknowledged ability, and much of his work stands to-day as a monument to his skill.

WHEREAS, in the death of Dr. Edgar Park, our esteemed associate and active worker, the St. Louis Dental Society has lost one of its most worthy and honored members, a man of sterling professional integrity, full of generous impulses and kindly feeling, therefore be it—

*Resolved*, That this expression of our regard and deep regret be spread upon the records of this Society, and that a copy be transmitted to the bereaved wife and family, to whom the society tender their sincere sympathy. Also—

*Resolved*, That a copy of this Memorial and Resolutions be sent to the leading Dental journals for publication.

WM. H. EAMES,

A. H. FULLER,

W. N. MORRISON,

*Committee.*

WM. CONRAD,

*Cor. Secretary.*

